# Changes in maternity care policies and practices that support breastfeeding as measured by the Ten Steps to Successful Breastfeeding — United States, 2018–2022

**DOI:** 10.1186/s12884-024-06672-z

**Published:** 2024-07-12

**Authors:** Kristin J. Marks, Lucas Gosdin, Lauren E. O’Connor, Heather C. Hamner, Daurice A. Grossniklaus

**Affiliations:** 1 Division of Nutrition, Physical Activity, and Obesity, Centers for Disease Control and Prevention, 4770 Buford Hwy, Atlanta, GA 30341 USA; 2 United States Public Health Service, 1101 Wootton Pkwy, Rockville, MD 20852 USA

**Keywords:** Breastfeeding, Maternity hospital, Postnatal care, Infant nutrition

## Abstract

**Background:**

Experiences during the birth hospitalization affect a family’s ability to establish and maintain breastfeeding. The Ten Steps to Successful Breastfeeding (Ten Steps) describe evidence-based hospital policies and practices shown to improve breastfeeding outcomes. We aim to describe hospitals’ implementation of the Ten Steps, changes over time, and hospitals’ implementation of a majority (≥ 6) of the Ten Steps by hospital characteristics and state.

**Methods:**

The biennial Maternity Practices in Infant Nutrition and Care (mPINC) survey assesses all hospitals in the United States (including the District of Columbia and territories) that routinely provide maternity care services. We analyzed data from 2018, 2020, and 2022 survey cycles to describe trends in the prevalence of hospitals implementing maternity care policies and practices that are consistent with the Ten Steps. Differences were calculated using the absolute difference in percentage-points between 2018 and 2022.

**Results:**

Between 2018 and 2022, the percentage of hospitals that implemented Step 2: Staff Competency and Step 5: Support Mothers with Breastfeeding increased 12 and 8 percentage points, respectively. The percentage of hospitals that implemented Step 6: Exclusive Breastfeeding Among Breastfed Infants was 7 percentage points lower in 2022 than 2018. Implementation of the remaining seven steps did not change by more than 5 percentage points in either direction between 2018 and 2022. Nationally, the percentage of hospitals that implemented ≥ 6 of the Ten Steps increased from 44.0% in 2018 to 51.1% in 2022. Differences were seen when comparing implementation of ≥ 6 of the Ten Steps by hospital characteristics including state, hospital size, and highest level of neonatal care offered.

**Conclusions:**

Nationally, maternity care policies and practices supportive of breastfeeding continued to improve; however, certain practices lost progress. Differences in implementation of the Ten Steps were observed across states and by certain hospital characteristics, suggesting more work is needed to ensure all people receive optimal breastfeeding support during their delivery hospitalization.

**Supplementary Information:**

The online version contains supplementary material available at 10.1186/s12884-024-06672-z.

## Introduction

The Dietary Guidelines for Americans, American Academy of Pediatrics, and World Health Organization (WHO) recommend that infants be exclusively breastfed for six months, after which complementary foods are introduced and breastfeeding continues for at least one (Dietary Guidelines for Americans [1]) or two years (American Academy of Pediatrics [2], WHO [3]). Among infants born in the United States in 2020, 83% of infants began breastfeeding; however, only a quarter were exclusively fed breast milk for the recommended first six months of life [4].

Low rates of exclusive and continued breastfeeding have public health implications. Infants who are breastfed are at a reduced risk for many acute and chronic pediatric conditions, such as otitis media, gastrointestinal infections, severe lower respiratory disease, sudden infant death syndrome (SIDS), type 1 diabetes, obesity, and asthma [5, 6]. There are also benefits to the breastfeeding mother, such as a reduced risk for breast cancer, ovarian cancer, type 2 diabetes, and hypertension [7]. The health effects from suboptimal breastfeeding rates contribute substantially to health care costs [8].

Although five in six U.S. mothers begin breastfeeding their infants, many do not continue breastfeeding as long as they intend [9]. Most U.S. infants are born in hospitals [10] and experiences during the birth hospitalization affect a parent-infant dyad’s ability to establish and maintain breastfeeding [11]. In 1991, the WHO and United Nations Children’s Fund (UNICEF) established the Baby-Friendly Hospital Initiative (BFHI) to implement the Ten Steps to Successful Breastfeeding (hereafter referred to as the Ten Steps) (Table S1) [12]. The Ten Steps describe evidence-based hospital policies and practices that have been shown to improve breastfeeding outcomes such as initiation, duration, and exclusivity [11, 13–16]. Studies show a dose-response association between the number of steps implemented and improved outcomes, suggesting that there are benefits to implementing any number of the Ten Steps [11, 14, 15]. For example, DiGirolamo et al. found an association between the number of steps of the Ten Steps a person experienced during their delivery hospitalization and longer breastfeeding duration: people who experienced none of the Ten Steps during their stay were 6.5 times more likely to stop breastfeeding before six weeks as those who experienced six steps [11].

Using data from the Maternity Practices in Infant Nutrition and Care (mPINC) survey, CDC has shown hospital adherence to the Ten Steps improved nationally between 2007 and 2013 [17, 18]; however, more work is needed to ensure all families receive optimal breastfeeding support during the birth hospitalization. Given that national data on hospital implementation of the Ten Steps has not been published in almost a decade, we use recent mPINC data to describe trends in hospital implementation of the Ten Steps from 2018 to 2022.

## Methods

### Data source

The Maternity Practices in Infant Nutrition and Care (mPINC) survey is conducted biennially by the U.S. Centers for Disease Control and Prevention [19]. All maternity care hospitals in the United States (including the District of Columbia and territories) are invited to participate. Hospitals report information on infant feeding policies and practices and routine maternity care. The mother–baby nurse manager or the manager of the labor and delivery unit is contacted to help identify the most knowledgeable staff person to receive the survey. The online survey is completed by the person(s) most knowledgeable about the hospital’s policies and practices related to infant nutrition.

Data for this analysis were obtained from the 2018, 2020, and 2022 survey cycles, which reflect policies and practices in the years 2017, 2019, and 2021, respectively. Response rates were 70% in 2018, 75% in 2020, and 72% in 2022.

### Variables

The mPINC Ten Steps Assessment Tool [20], which aligns the 2018–2022 mPINC survey questions with the updated 2018 BFHI Implementation Guidance [12], was used to identify mPINC survey questions related to each of the Ten Steps. Although the tool aligns mPINC survey questions with the Ten Steps, the mPINC survey cannot comprehensively assess each step; rather, it provides indicators related to each step. Within each of the Ten Steps indicators, there are one or more items that contribute to the indicator. Items may be defined by multiple mPINC survey questions. All responses were dichotomized (implementing all ideal standards or not implementing all ideal standards) based on the mPINC Ten Steps Assessment Tool. Hospitals were then categorized as either “implementing the Ten Steps indicator” if they reported the ideal standard for each survey item related to the respective step or as “not implementing the Ten Steps indicator” if at least one of the responses was not the ideal standard. The outcomes of this analysis were indicators of implementation for each of the Ten Steps and the total number of the Ten Steps implemented.

The total number of steps implemented by each hospital was calculated, with possible values ranging from zero to ten steps implemented. We then created a dichotomous variable defined as implementing a majority of the Ten Steps (≥ 6 steps) or not implementing a majority of the Ten Steps (< 6 steps).

Hospital characteristics were considered covariates and included the following self-reported variables: hospital ownership type (government/military; nonprofit, private; or for profit, private), teaching hospital (yes or no), hospital size as defined by the number of annual births (< 250, 250–499, 500–999, 1000–1999, 2000–4999, or ≥ 5000 births), and highest level of neonatal care (Level I: Well newborn nursery, Level II: Special care nursery, Level III: Neonatal Intensive Care Unit (NICU), or Level IV: Regional NICU). Geographic variables including state, region, and rurality were also considered covariates and were defined based on the hospital’s confirmed address. The seven regions (Western, Mountain Plains, Southwest, Midwest, Southeast, Mid-Atlantic, or Northeast) were defined by the U.S. Department of Agriculture’s (USDA) Food and Nutrition Service [21]. Rurality was defined using USDA’s Economic Research Service’s Rural-Urban Continuum Codes to classify hospitals as being located in metro or non-metro counties [22].

### Statistical analysis

Ten Step indicators (e.g., Step 1: Hospital Policies) are based on one or more items (Table S1). Items (e.g., Compliance with the Code) can be comprised of multiple mPINC survey questions [20]. Items were coded as missing if half or more of the survey questions that made up the item were missing data. Ten Step indicators were coded as missing if half or more of the items that make up the step were missing. We removed hospitals with missing data on Ten Steps indicators from the analysis (*n* = 7, *n* = 7, *n* = 3 in 2018, 2020, and 2022, respectively). This resulted in 2,038 participating hospitals in 2018; 2,096 participating hospitals in 2020; and 1,991 participating hospitals in 2022 included in our analytic dataset.

**Table 1:** Percentage of hospitals with the ideal policy or practice on each item of the Ten Steps to Successful Breastfeeding, CDC’s Maternity Practices in Infant Nutrition and Care (mPINC) survey, by year — United States, 2018–2022.^﻿a^

Ten Steps to Successful Breastfeeding	mPINC measure^b^	% with ideal response			Difference (2022 − 2018)
		2018 *n* = 2038	2020 *n* = 2096	2022 *n* = 1991	
**Step 1: Hospital Policies** ^**c**^		**23.3%**	**25.8%**	**26.5%**	**3.2**
Compliance with the Code^d^	Acquisition of infant formula and no distribution of infant formula or formula-related supplies/coupons as gifts	37.1%	41.6%	44.4%	7.3
Written infant feeding policies	Written policies	28.6%	32.4%	31.7%	3.1
Monitoring and data-management systems	Documentation of exclusive breastfeeding	92.9%	91.8%	92.2%	-0.7
**Step 2: Staff competency**		**36.5%**	**36.1%**	**48.7%**	**12.2**
Staff competency to support breastfeeding	Nurse skill competency	60.9%	67.9%	72.7%	11.8
Assessment of staff competency to support breastfeeding	Nurse competency assessment	46.6%	41.8%	57.0%	10.4
**Step 3: Prenatal Care**		**95.8%**	**93.8%**	**92.0%**	**-3.8**
Prenatal breastfeeding education	Prenatal breastfeeding education				
**Step 4: Care Right After Birth**		**47.8%**	**50.5%**	**48.5%**	**0.7**
Immediate skin-to-skin contact after vaginal delivery	Immediate skin-to-skin contact after vaginal delivery	66.9%	70.8%	69.9%	3.0
Immediate skin-to-skin contact after Cesarean-delivery	Immediate skin-to-skin contact after Cesarean-delivery	49.3%	51.7%	49.9%	0.6
**Step 5: Support Mothers with Breastfeeding**		**66.0%**	**69.7%**	**74.4%**	**8.4**
Practical support to initiate and maintain breastfeeding and manage common breastfeeding problems	Identify/solve breastfeeding problems				
**Step 6: Exclusive Breastfeeding Among Breastfed Infants**		**24.2%**	**24.2%**	**17.1%**	**-7.1**
Non-medically indicated supplementation	No formula-feeding of breastfed infants unless medically indicated	38.9%	37.6%	28.3%	-10.6
Counseling on the importance of exclusive breastfeeding	Formula counseling for breastfeeding mothers	60.4%	61.2%	59.3%	-1.1
Instruction of formula feeding techniques and safe preparation and handling of formula	Formula preparation and feeding techniques	70.9%	75.9%	78.0%	7.1
**Step 7: Rooming-in**		**18.9%**	**24.0%**	**23.6%**	**4.7**
Mother-infant separation after birth	Transition	85.6%	89.4%	89.9%	4.3
Rooming-in for 24 h/day	Rooming-in	73.4%	81.3%	84.1%	10.7
Mother-infant separation while rooming-in	Limit mother-infant separation	27.1%	32.3%	33.0%	5.9
Observation of mother-infant dyads to ensure safety	Rooming-in safety	71.1%	76.0%	74.6%	3.5
**Step 8: Responsive Feeding**		**87.8%**	**89.8%**	**92.1%**	**4.3**
Support recognition and response to infant feeding cues	Feeding cues				
**Step 9: Bottles**,** Teats (Nipples)**,** and Pacifiers**		**75.2%**	**76.8%**	**77.3%**	**2.1**
Counseling on the risks of artificial teats (nipples) and pacifiers	No pacifiers				
**Step 10: Discharge**		**39.1%**	**41.7%**	**39.2%**	**0.1**
Coordination of discharge to ensure appropriate follow-up care	Post-discharge follow-up visit	86.5%	87.7%	88.8%	2.3
Coordination of discharge to ensure ongoing breastfeeding support	Post-discharge breastfeeding support	43.7%	45.8%	42.8%	-0.9

^a^ See mPINC Ten Steps Assessment Tool, which is a crosswalk aligning mPINC and the Ten Steps [20]

^b^ See mPINC Scoring Algorithm for detailed descriptions of mPINC measures and their ‘ideal’ responses [23]

^c^ Ten Step indicators (the bolded headers) were coded as missing if half or more of the items that made up the step were missing

^d^ Items (rows below the bolded Ten Step indicators) can be comprised of multiple mPINC survey questions. Ten Step indicators are based on one or more items. Items were coded as missing if half or more of the survey questions that made up the item were missing data

We described the percent of hospitals that implemented each item and step by survey cycle. Second, we plotted the percent of hospitals by number of the Ten Steps implemented stratified by survey cycle. Third, we presented the percent of hospitals that implemented a majority (≥ 6) of the Ten Steps by hospital characteristic (e.g., size, region) by survey cycle. Lastly, we mapped the percent of hospitals that implemented a majority (≥ 6) of the Ten Steps by state stratified by survey cycle. Data were not mapped for the District of Columbia, Rhode Island, Guam, American Samoa, the Northern Mariana Islands, or the U.S. Virgin Islands due to small sample size (< 5 hospitals). Differences were calculated using the absolute difference in percentage-points between 2018 and 2022.

No statistical tests were performed because data were obtained from a census of hospitals providing routine maternity care, therefore, there was no sampling error. Analyses were conducted using SAS 9.4 (SAS Institute Inc., Cary, NC). This activity was reviewed by the Centers for Disease Control and Prevention (CDC), deemed not research – public health surveillance, and was conducted consistent with applicable federal law and CDC policy (See e.g., 45 C.F.R. part 46.102(l)(2), 21 C.F.R. part 56; 42 U.S.C. § 241(d); 5 U.S.C. § 552a; 44 U.S.C. § 3501 et seq.).

## Results

In 2022, the most implemented of the Ten Steps were Step 3: prenatal breastfeeding education (92.0%) and Step 8: responsive feeding (92.1%) (Table 1); both steps are comprised of one item. The least implemented of the Ten Steps in 2022 were Step 6: Exclusive Breastfeeding Among Breastfed Infants (17.1%) and Step 7: Rooming-in (23.6%); both steps are comprised of three or more items.

Substantial progress (defined as ≥ 5 percentage points higher) was seen between 2018 and 2022 in the proportion of hospitals that implemented Step 2: Staff Competency (36.5 to 48.7%) and Step 5: Support Mothers with Breastfeeding (66.0 to 74.4%). A substantial decline (defined as ≥ 5 percentage points lower) occurred in Step 6: Exclusive Breastfeeding Among Breastfed Infants (24.2 to 17.1%). Of the three items that comprise Step 6, the percentage of hospitals with the ideal response regarding non-medically indicated supplementation of breastfed infants worsened from 2018 to 2022; 28.3% of hospitals did not routinely feed formula to healthy, breastfed infants when there was no medical reason to do so in 2022, down from 38.9% in 2018. Hospitals with ideal responses for formula counseling for breastfeeding parents held steady (60.4 to 59.3%) and instruction of formula feeding techniques and safe preparation and handling of formula for parents feeding any formula improved (70.9 to 78.0%).

**Fig. 1:**
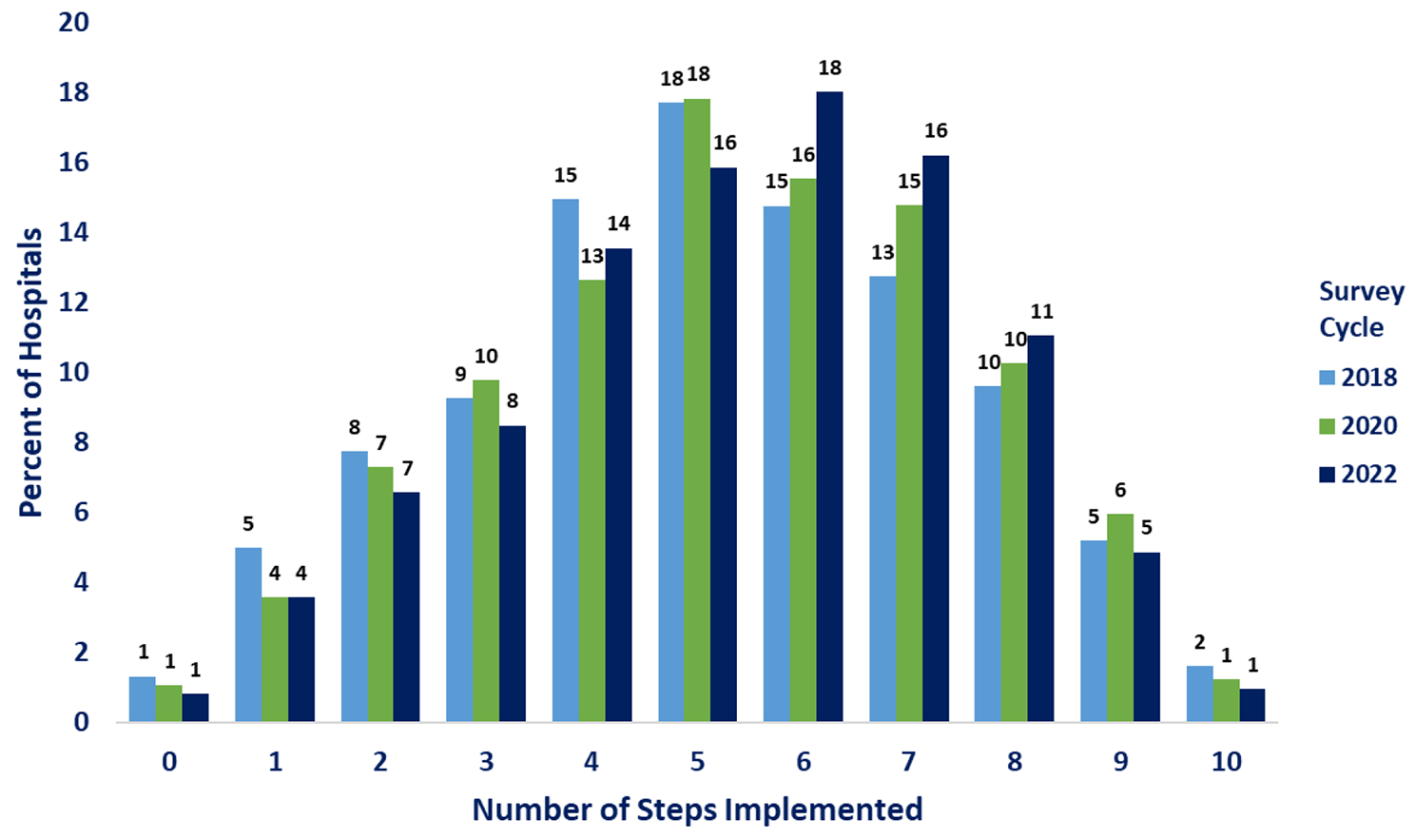
Percentage of hospitals implementing the Ten Steps to Successful Breastfeeding by number of steps implemented — Maternity Practices in Infant Nutrition and Care (mPINC) Survey, United States, 2018–2022

Nationally, 44.0% of hospitals implemented a majority (≥ 6) of the Ten Steps in 2018, while 51.1% of hospitals did so in 2022 (Fig. 1; Table 2). Comparing 2022 to 2018, the distribution of hospitals has shifted towards more steps implemented; however, the proportion of hospitals implementing the highest number of the Ten Steps (i.e., 9 or 10 steps) has decreased slightly. The most prevalent number of steps implemented in 2022 was six steps (18.0%) followed by seven steps (16.2%), whereas in 2018, it was five steps (17.7%) followed by four steps (15.0%).

**Table 2:** Characteristics of hospitals implementing more than half (≥ 6) of the Ten Steps to Successful Breastfeeding, CDC’s Maternity Practices in Infant Nutrition and Care (mPINC) survey, by year — United States, 2018–2022

	2018	2020	2022
Hospital Characteristic	Hospitals Implementing ≥ 6 of the Ten Steps	Hospitals Implementing ≥ 6 of the Ten Steps	Hospitals Implementing ≥ 6 of the Ten Steps
	*N* = 2,038 *n* (%)^a^	*N* = 2,096 *n* (%)^a^	*N* = 1,991 *n* (%)^a^
**Total**	896 (44.0)	1002 (47.8)	1018 (51.1)
**Hospital Ownership Type**			
Government/Military	45 (48.9)	132 (48.7)	125 (52.1)
Non-profit, private	714 (45.7)	743 (48.8)	766 (52.6)
For profit, private	137 (35.7)	127 (42.1)	127 (43.2)
**Teaching Hospital**			
Yes	618 (43.9)	762 (49.2)	775 (51.5)
No	278 (44.2)	240 (44.0)	243 (50.0)
**Hospital Size (annual births)**			
< 250	123 (36.6)	139 (37.9)	130 (36.8)
250–499	139 (36.7)	155 (40.2)	168 (47.9)
500–999	184 (42.3)	227 (50.3)	227 (52.2)
1000–1999	219 (48.8)	235 (53.0)	247 (57.4)
2000–4999	199 (51.2)	216 (54.3)	213 (58.4)
≥ 5000	32 (64.0)	30 (62.5)	30 (60.0)
**Highest Level of Neonatal Care**			
Level I: Well newborn nursery	316 (38.9)	353 (42.2)	337 (42.9)
Level II: Special care nursery	272 (43.0)	302 (47.3)	330 (54.5)
Level III: Neonatal Intensive Care Unit	263 (52.4)	290 (55.2)	299 (58.7)
Level IV: Regional Neonatal Intensive Care Unit	44 (51.8)	53 (58.9)	50 (59.5)
**Region**			
Northeast	101 (54.6)	115 (59.0)	114 (61.6)
Western	145 (53.9)	146 (50.5)	148 (53.8)
Mid-Atlantic	90 (40.2)	104 (49.1)	103 (51.8)
Midwest	212 (43.4)	229 (46.8)	223 (49.6)
Southwest	131 (40.6)	151 (46.2)	156 (48.8)
Mountain Plains	92 (41.1)	111 (45.3)	108 (46.8)
Southeast	125 (38.5)	146 (43.1)	166 (50.2)
**Rurality** ^**b**^			
Rural	235 (34.7)	273 (38.9)	273 (41.0)
Urban	661 (48.6)	729 (52.3)	745 (56.2)

^a^ Row percents shown

^b^ Defined using USDA’s Economic Research Service’s Rural-Urban Continuum Codes to classify metro and non-metro counties [22]

Across survey cycles, government/military and non-profit, private hospitals were more likely to implement a majority of the Ten Steps than for profit, private hospitals. For example, in 2022, 52.1% of government/military and 52.6% of non-profit, private hospitals implemented a majority of the Ten Steps, compared to 43.2% of for profit, private hospitals (Table 2). Little difference in implementation of a majority of the Ten Steps was observed by teaching hospital status. There was a positive association observed between hospital size and the proportion of hospitals implementing a majority of the Ten Steps, with hospitals with more annual births being more likely to implement. Similarly, there was a positive association observed with level of neonatal care, with Level IV (regional NICU) being the most likely to implement a majority of the Ten Steps.

**Fig. 2:**
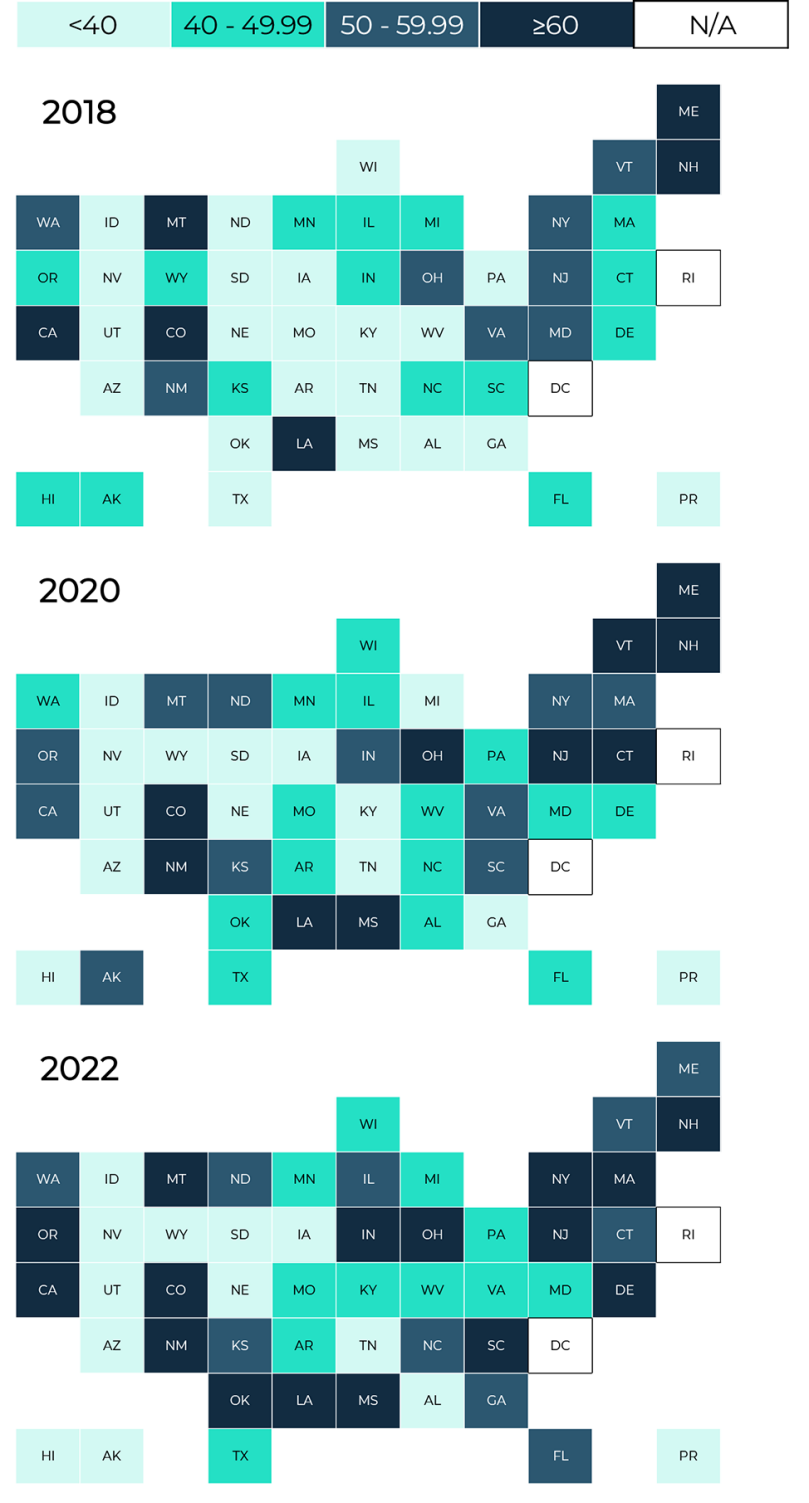
Percentage of hospitals implementing more than half (≥ 6) of the Ten Steps to Successful Breastfeeding, by state or territory — Maternity Practices in Infant Nutrition and Care (mPINC) Survey, United States, 2018–2022

In terms of geography, hospitals located in the Northeast and Western regions were most likely to implement a majority of the Ten Steps across survey cycles (Northeast: 61.6% and Western: 53.8% in 2022) (Table 2). More urban hospitals implemented a majority of the Ten Steps than rural hospitals (56.2% vs. 41.0% in 2022). There was substantial variation in implementation of the Ten Steps by state (Fig. 2). Eleven states had half or more of their hospitals (≥ 50%) implementing a majority of the Ten Steps in 2018, 2020, and 2022; those states were: California, Colorado, Louisiana, Maine, Montana, New Hampshire, New Jersey, New Mexico, New York, Ohio, and Vermont.

## Discussion

As of 2022, more than half of U.S. hospitals are implementing a majority (≥ 6) of the Ten Steps. From 2018 to 2022, several key aspects of maternity care supportive of breastfeeding have shown consistent improvement, while a few practices have declined. Differences in implementation of the Ten Steps were observed across states and by certain hospital characteristics, such as size and highest level of neonatal care offered. Taken together, these findings suggest more work is needed to ensure all families receive optimal breastfeeding support during the birth hospitalization.

Following every survey cycle, individualized mPINC reports are sent to each participating hospital. These reports show specific areas of care where changes can be made to support breastfeeding families. In key informant interviews, staff from participating hospitals described using mPINC reports for quality improvement initiatives, benchmarking, and staff education (unpublished data), all of which might contribute to improved hospital policies and practices. Further, dissemination of surveillance data from the mPINC survey, such as state reports [24], might be a factor in improvements in maternity care practices. Some state-specific recognition programs acknowledge hospitals for implementing a portion or all the Ten Steps. Several states use mPINC data, along with other breastfeeding data, to inform their recognition programs [19].

In the 2018, 2020, and 2022 survey cycles, there were a few steps less commonly implemented by hospitals, namely Step 1: Hospital Policies (26.5% in 2022), Step 7: Rooming-in (23.6% in 2022), and Step 6: Exclusive Breastfeeding Among Breastfed Infants (17.1% in 2022). Within Step 1, most hospitals have monitoring and data management systems in place to document exclusive breastfeeding (92.2% in 2022). Beginning in 2024, the Centers for Medicare and Medicaid Services removed the Perinatal Care (PC) electronic clinical quality measure ePC-05 (Exclusive Human Milk Feeding) from the Inpatient Quality Reporting Program [25]. The Joint Commission will maintain the ePC-05 measure for optional use, but it remains to be seen how this change in reporting requirements will impact hospital monitoring and data management systems used to document exclusive breastfeeding. Also within Step 1, fewer hospitals comply with the International Code of Marketing of Breastmilk Substitutes (44.4% in 2022) [26] and have comprehensive written feeding policies (31.7% in 2022). The International Code of Marketing of Breastmilk Substitutes encourages hospitals to purchase formula and feeding devices at fair market value. Implementation of hospital policy for market price purchasing of infant formula is associated with reduced in-hospital formula supplementation and increased in-hospital exclusive breastfeeding and breastfeeding duration [27]. More broadly, written hospital breastfeeding policies are associated with increased breastfeeding [15, 28]. For hospitals looking to implement comprehensive written feeding policies supportive of breastfeeding and the Ten Steps, the Academy of Breastfeeding Medicine provides an example [29] that hospitals can use as a model for their own institutional policies.

Within Step 7, few hospitals limit mother-infant separation (33.0% in 2022). To be considered adherent to this item, the usual location of newborns during (1) pediatric exams/rounds, (2) hearing screening, (3) pulse oximetry screening, (4) routine labs/blood draws/injections, and (5) newborn bath should be in the mother’s room. According to guidance, all routine procedures, assessments, screenings, immunizations, and laboratory draws can be performed during skin-to-skin contact or at the mother’s bedside to minimize mother-infant separation [30–32]. Research suggests that early separation may interfere with mother-infant interaction during breastfeeding [33]. As these findings suggest, limiting mother-infant separation remains an area of opportunity for many hospitals.

In addition to an already low prevalence of implementation, hospitals got worse on Step 6: Exclusive Breastfeeding Among Breastfed Infants, from 24.2% implementation in 2018 to 17.1% in 2022. The non-medically indicated supplementation item worsened from 38.9% in 2018 to 28.3% in 2022, largely driving the decline seen in the Step 6 indicator. This item assesses the percentage of hospitals that provide infant formula to healthy, breastfed infants without medical reason to do so. Surveillance data from the National Immunization Survey-Child corroborate this trend, showing that formula supplementation of breastfed infants before two days of age is increasing in prevalence, from 15.5% among infants born in 2014 to 20.8% among infants born in 2020 [4]. It is well-documented that non-medically indicated formula supplementation during the birth hospitalization disrupts early breastfeeding [34, 35] and recent work demonstrates that the supplementation of breastfed infants with formula before two days of age is significantly associated with shorter breastfeeding duration [36]. These findings reinforce the influence and value of maternity care practices on long-term breastfeeding and draw attention to increased non-medically indicated formula supplementation of breastfed infants before two days.

Throughout the period under examination, there were two steps implemented by most hospitals. Specifically, hospitals reported strong adherence to Step 3: Prenatal Care (prenatal breastfeeding education, 92.0% in 2022) and Step 8: Responsive Feeding (recognizing and responding to infant feeding cues, 92.1% in 2022). Both Steps 3 and 8 focus on the education of new parents about breastfeeding, prior to and during the birth hospitalization. Studies show that breastfeeding education and support improve breastfeeding initiation and duration [37, 38]. Hospitals can continue to emphasize breastfeeding education to set families up for success in breastfeeding.

In this study, we observed substantial variation in implementation of the Ten Steps by hospital characteristics such as state, ownership type, size, and highest level of neonatal care offered. Previous work has identified characteristics of hospitals that were more likely to be Baby-Friendly: urban, Northeast region, high number of births, or not for-profit [39]. The findings of the present study examining implementation of a majority of the Ten Steps align with these results and suggest the highest level of neonatal care offered as another predictor. Geographic location, as indicated by state, is also associated with implementation of a majority of the Ten Steps, as previous work has also shown [17]. Various state-level policies [40] and initiatives [41] could influence implementation of the Ten Steps by state. Recent work has examined geographic disparities in exclusive in-hospital breastfeeding by BFHI designation [42, 43].

While it is not possible to understand the total effect of the COVID-19 pandemic on changes seen in the prevalence of practices and policies supportive of breastfeeding, there is the potential that both improvements and declines in certain practices during this period were impacted by changes brought about by the pandemic. For example, there was a decline of almost 4 percentage points in prenatal breastfeeding education between 2018 (95.8%) and 2022 (92.0%), which could be related to the challenges of meeting in person that arose during the pandemic. Similarly, rooming-in practices improved dramatically between 2018 (73.4%) and 2022 (84.1%), which may be due in part to isolating family units to avoid potential COVID-19 exposure. Data from the summer of 2020 show that hospitals changed their practices in response to the COVID-19 pandemic: 18% of hospitals reported decreased in-person lactation support and 73% reported discharging parents and their babies < 48 h after birth [44]. It is unclear the extent to which changes between 2018 and 2022 can be attributed solely to the COVID-19 pandemic, but it is worth noting that these data cover an extraordinary period when changes were being made in response to the pandemic, among other reasons.

This study has notable strengths: mPINC is a census of all maternity hospitals and has a high response rate. mPINC data are used for national surveillance, public health research, state-level public health practice, and individual hospital improvement. The findings in this report are subject to at least three limitations. First, the survey is completed by a key respondent on behalf of each hospital which might limit the accuracy of the hospital practices assessed and demographics reported. However, to ensure that the data obtained were of the highest quality possible, CDC requested that the survey be sent to the person(s) most knowledgeable about the hospital’s maternity practices, and that it be completed in consultation with other knowledgeable persons. Second, although 70–75% of hospitals participated in each survey, practices might differ between those that participated and those that did not. State level percentages may vary across survey cycles depending on the number of hospitals that participated in each survey cycle, and this can be more pronounced among states with a small number of hospitals. Third, the mPINC survey questions used are consistent with the Ten Steps to Successful Breastfeeding but do not indicate implementation of each of the steps as required for designation by BFHI. While mPINC assesses many aspects of each of the Ten Steps, it does not assess every aspect.

## Conclusions

The national improvements in maternity care supportive of breastfeeding from 2018 to 2022 are notable. However, these data also indicate variation in the implementation of certain steps. The findings highlight areas where more focused attention may be needed to ensure all families have access to evidence-based maternity care policies and practices supportive of breastfeeding [17].

### Electronic supplementary material

Below is the link to the electronic supplementary material.

Supplementary Material 1

## Data Availability

Data are available upon request to mpinc@cdc.gov.

## Abbreviations

BFHI: Baby-Friendly Hospital Initiative
CDC: Centers for Disease Control and Prevention
mPINC: Maternity Practices in Infant Nutrition and Care
NICU: Neonatal Intensive Care Unit
Ten Steps: Ten Steps to Successful Breastfeeding
UNICEF: United Nations Children’s Fund
USDA: U.S. Department of Agriculture
WHO: World Health Organization
