# Supplementary material for: Changes in maternity care policies and practices that support breastfeeding as measured by the Ten Steps to Successful Breastfeeding — United States, 2018–2022
Source: BMC Pregnancy Childbirth. 2024 Jul 12;24:475. doi: 10.1186/s12884-024-06672-z (PMC11241842; doi:10.1186/s12884-024-06672-z)
Supplement: Supplementary file 1 — Supplementary Material 1 [file 12884_2024_6672_MOESM1_ESM.docx]

**Table S1.** The Ten Steps to Successful Breastfeeding (revised 2018) [12]

| **Critical management procedures** |
| --- |
| 1. a. Comply fully with the *International Code of Marketing of Breast-milk Substitutes* and relevant World Health Assembly resolutions. |
| 1. b. Have a written infant feeding policy that is routinely communicated to staff and parents. |
| 1. c. Establish ongoing monitoring and data-management systems. |
| 2. Ensure that staff have sufficient knowledge, competence and skills to support breastfeeding. |
| **Key clinical practices** |
| 3. Discuss the importance and management of breastfeeding with pregnant women and their families. |
| 4. Facilitate immediate and uninterrupted skin-to-skin contact and support mothers to initiate breastfeeding as soon as possible after birth. |
| 5. Support mothers to initiate and maintain breastfeeding and manage common difficulties. |
| 6. Do not provide breastfed newborns any food or fluids other than breast milk, unless medically indicated. |
| 7. Enable mothers and their infants to remain together and to practice rooming-in 24 hours a day. |
| 8. Support mothers to recognize and respond to their infants’ cues for feeding. |
| 9. Counsel mothers on the use and risks of feeding bottles, teats and pacifiers. |
| 10. Coordinate discharge so that parents and their infants have timely access to ongoing support and care. |
